# Does Prior Percutaneous Coronary Intervention Influence the Outcomes of Coronary Artery Bypass Surgery?

**DOI:** 10.21470/1678-9741-2019-0234

**Published:** 2020

**Authors:** Gade S. V. Miguel, Alexandre G. Sousa, Gilmara S. Silva, Flávia C. Colósimo, Noedir A. G. Stolf

**Affiliations:** 1 Cardiothoracic Surgeon, Clínica Girassol, Luanda, Angola.; 2 Clinical Research Physician, Hospital Beneficência Portuguesa, São Paulo, SP, Brazil.; 3 Research Nurse, Center for Education and Research of the Hospital Beneficência Portuguesa, São Paulo, SP, Brazil.; 4 University of São Paulo Medical School, São Paulo, SP, Brazil.

**Keywords:** Coronary Artery Disease, Propensity Score, Patient Readmission, Coronary Artery Bypass, Percutaneous Coronary Intervation, Registries

## Abstract

**Introduction:**

Percutaneous coronary intervention (PCI) has been increasingly performed to treat coronary artery disease. The performance of multiple PCI has also been increasing. Consequently, the percentage of patients presenting for coronary artery bypass graft (CABG) surgery is reported to vary from 13 to 40%. The influence of previous PCI on CABG outcomes has been studied in single center, regional studies, registries and meta-analyses. Some reports showed a negative effect on mortality and morbidity in early or long-term follow-up, but others did not find this influence.

**Methods and Results:**

A cohort of 3007 patients consecutively operated for CABG, 261 of them with previous PCI, were included in this analysis. Comparison of the groups "previous PCI" and "primary CABG" was made in the original cohort and in a propensity score matched cohort of 261 patients. There were some differences in preoperative clinical characteristics in both types of cohort, even in the matched one. Outcomes were compared at 30 days, 1 year and 5 years of follow-up. There were no statistically significant differences in mortality in any period or cohort. There were some differences in other outcomes as readmission and composite events, including cardiovascular death at 1 and 5 years of follow-up. These differences, neverthless, were not confirmed in comparison with the matched cohort.

**Conclusion:**

Although there are some limitations in this study, it was not found consistent negative influence of previous PCI on CABG.

**Table:** 

Abbreviations, acronyms & symbols	
ANOVA	= Analysis of variance
CABG	= Coronary artery bypass grafting
E-CABG	= European Multicenter Study on Coronary Artery Bypass Grafting
MACCE	= Major adverse cerebral and cardiovascular events
PCI	= Percutaneous coronary intervention
REVASC	= Registry of Revascularization of Myocardium
STS	= Society of Thoracic Surgeons

## INTRODUCTION

European and American guidelines recommend surgery for multivessel and complex left main coronary artery disease. Neverthless, several factors, including improved coronary stent technology and the less invasive nature of PCI, has led to a continued increase in the use of this therapy. Furthermore, there is also an increase in multiple PCI procedures. Consequently, the percentage of patients presenting for CABG with previous PCI has increased and is reported to range from 13 to 40%^[1]^. Since 2005, single center, regional studies, registries and systematic reviews and meta-analyses have investigated the potential adverse effect of previous PCI on CABG. Results are controversial^[1-29]^.

## METHODS

This is a retrospective study using the REVASC (Registry of Revascularization of Myocardium) database, of Hospital Beneficência Portuguesa - São Paulo, with 3007 consecutive patients operated on from June 2009 to July 2010. Comparison of mortality and major cardiovascular and neurological events in previous PCI and no previous PCI groups in the 3007 original cohort and in propensity score matching group of 261 patients was performed. Data were analyzed at 30 days, 1 year and 5 years of follow-up in both cohorts.

Patient characteristics of each group in both cohorts and risk factors were performed according to EuroSCORE definitions. The endpoints in this study were adapted according to the Society of Thoracic Surgeons (STS) definitions of mortality and major adverse cerebral and cardiovascular events (MACCE): mortality as death of any cause and mortality of cardiovascular cause in the 3 defined follow-up periods and MACCE as the occurence of one or a combination of the following events: death of cardiovascular cause, new nonfatal myocardial infarction, transient or permanent cerebrovascular event and need for readmission (Tables 1 and 2).

**Table 1:** Clinical outcomes in 30 days, 1 and 5 years. Original cohort.

Variable	PCI		*P*- value	OR	95% CI
	**No**	**Yes**			
Death in 30 days	120 (4.4)	9 (3.5)	0.483^(^1^)^	0.78	(0.39-1.56)
CVD death in 30 days	7 (0.3)	2 (0.8)	0.181^(^2^)^	3.01	(0.62-14.58)
MI in 30 days	33 (1.2)	1 (0.4)	0.359^(^2^)^	0.32	(0.04-2.32)
Stroke in 30 days	51 (1.9)	2 (0.8)	0.320^(^2^)^	0.41	(0.10-1.69)
Death/MI/stroke in 30 days	86 (3.1)	5 (1.9)	0.270^(^1^)^	0.60	(0.24-1.50)
Death in 1 year	237 (8.6)	19 (7.3)	0.455 ^(^1^)^	0.83	(0.51-1.35)
CVD death in 1 year	55 (2.0)	7 (2.7)	0.472^(^1^)^	1.34	(0.60-2.97)
Readmission for any cause in 1 year	498 (18.3)	61 (23.5)	0.045^(^1^)^	1.37	(1.01-1.85)
CV readmission for 1 year	251 (9.2)	35 (13.5)	0.028^(^1^)^	1.53	(1.05-2.23)
Death/CV readmission for 1 year	251 (9.2)	35 (13.5)	0.028^(^1^)^	1.53	(1.05-2.23)
Death in 5 years	408 (17.0)	35 (14.8)	0.376^(^1^)^	0.85	(0.58-1.23)
CVD death in 5 years	134 (5.6)	13 (5.5)	0.946^(^1^)^	0.98	(0.55-1.76)
Readmission for any cause in 5 years	848 (35.4)	102 (43)	0.019^(^1^)^	1.38	(1.05-1.81)
CV readmission in 5 years	359 (15.0)	47 (19.8)	0.048^(^1^)^	1.41	(1.00-1.97)
Death/CV readmission in 5 years	399 (16.7)	51 (21.5)	0.057^(^1^)^	1.37	(0.99-1.91)

(1) Descriptive probability level of Student’s t-test.

(2) Descriptive probability level of chi-square test

(3) Descriptive probability level of Fisher's exact test.

(4) Descriptive probability level of Mann-Whitney nonparametric test.

CV=cardiovascular; CVD=cardiovascular disease; CI=confidence interval; MI=myocardial infarction; OR=odds ratio

**Table 2:** Clinical outcomes in 30 days, 1 and 5 years. Matched cohort.

Variable	PCI		*P*- value	Odds ratio	95% CI
	**No.**	**Yes**			
Death in 30 days	11 (4.2)	9 (3.5)	0.648^(^1^)^	0.81	(0.33-1.99)
CVD death in 30 days	2 (0.8)	2 (0.8)	1.000(2)	1.00	(0.14-7.13)
MI in 30 days	4 (1.5)	1 (0.4)	0.373(2)	0.25	(0.03-2.23)
Stroke in 30 days	3 (1.2)	2 (0.8)	1.000(2)	0.66	(0.11-4.00)
Death/MI/stroke in 30 days	8 (3.1)	5 (1.9)	0.396^(^1^)^	0.62	(0.20-1.91)
Death in 1 year	22 (8.4)	19 (7.3)	0.626^(^1^)^	0.85	(0.45-1.62)
CVD death in 1 year	9 (3.5)	7 (2.7)	0.599^(^1^)^	0.78	(0.28-2.07)
Readmission for any cause in 1 year	55 (21.5)	61 (23.5)	0.591^(^1^)^	1.12	(0.74-1.69)
CV readmission in 1 year	27 (10.6)	35 (13.5)	0.309^(^1^)^	1.32	(0.77-2.25)
Death/CV readmission in 1 year	27 (10.6)	35 (13.5)	0.309^(^1^)^	1.32	(0.77-2.25)
Death in 5 years	37 (17.0)	35 (14.8)	0.520^(^1^)^	0.85	(0.51-1.40)
CVD death in 5 years	17 (7.8)	13 (5.5)	0.321^(^1^)^	0.69	(0.33-1.45)
Readmission for any cause in 5 years	79 (36.2)	102 (43.0)	0.139^(^1^)^	1.33	(0.91-1.94)
CV readmission in 5 years	31 (14.2)	47 (19.8)	0.113^(^1^)^	1.49	(0.91-2.45)
Death/CV readmission in 5 years	40 (18.4)	51 (21.5)	0.398^(^1^)^	1.22	(0.78-1.94)

(1) Descriptive probability level of Student’s t-test.

(2) Descriptive probability level of chi-square test.

(3) Descriptive probability level of Fisher's exact test.

(4) Descriptive probability level of nonparametric Mann-Whitney test.

CV=cardiovascular; CVD=cardiovascular disease; CI=confidence interval; MI=myocardial infarction; OR=odds ratio

In statistics, initially a descriptive analysis of absolute and relative frequency, mean and standard deviation of the variables of interest was performed. Pearson’s chi-square test or Fisher’s exact test were used to verify equality of proportions between the interest groups when variables were qualitative. For the comparison of quantitative variables we used the Student’s t-test or the nonparametric Mann-Whitney test for 2 groups or the ANOVA or nonparametric Kruskal-Wallis test for 3 or more groups. Some multivariable regression models as linear or logistic regression were performed for survival analysis. To control the bias of a nonrandomized study, a propensity score matching model was constructed (this is considered a very powerful tool used in this type of study for the reason mentioned). In the “no previous PCI” group, we selected cases who were similar to patients in the “previous PCI” group. For this matching score we selected age, gender, diabetes, dyslipidemia, heart failure, previous myocardial infarction and unstable angina as factors for the matching.

Data analysis was performed using SPSS Statistics software for Windows, version 16.0.

All tests were made considering bilateral hypothesis and assuming significance level ≤5%.

## RESULTS

Patients were classified into 2 groups: in the original cohort, 2746 patients without previous PCI and 261 patients with previous PCI; in the the propensity score matching, 261 patients in each group.

### Preoperative Clinical Characteristics

Preoperative data are shown in Tables 3 and 4. In the original cohort, there were statistically significant differences regarding age (PCI younger patients); dyslipidemia (more frequent in PCI patients); peripheral artery disease (more in the PCI group); previous myocardial infarction (more frenquent in the PCI group); EuroSCORE (higher in the non-PCI group) and nonelective surgery (more frequent in the PCI group). In the propensity score matching group, only dyslipidemia and peripheral artery insufficiency remained different.

**Table 3:** Preoperative clinical characteristics. Original cohort.

Variable		(n=3007)PCI		*P*-value
		**No.** **(n=2746)**	**Yes** **(n=261)**	
Median age (years)		62.3±9.4	61.0±10.0	0.032^(^1^)^
Male gender		1911 (69.6)	192 (73.6)	0.181^(^2^)^
Race	White	2316 (84.3)	228 (87.4)	
	Black	114 (4.2)	7 (2.7)	0.515^(^2^)^
	Asians	281 (10.2)	24 (9.2)	
	Others	35 (1.3)	2 (0.8)	
BMI	<25	913 (34.2)	87 (34.8)	
	25-30	1212 (45.4)	106 (42.4)	0.562^(^2^)^
	≥30	543 (20.4)	57 (22.8)	
Smoking	Previous	1095 (39.9)	107 (41.0)	
	Never	1221 (44.5)	123 (47.1)	0.262^(^2^)^
	Current	430 (15.7)	31 (11.9)	
Family history of CVD		800 (29.1)	80 (30.7)	0.607^(^2^)^
Diabetes mellitus		995 (36.2)	106 (40.6)	0.161^(^2^)^
Dyslipidemia		1195 (43.5)	142 (54.4)	<0.001^(^2^)^
Chronic kidney failure		157 (5.7)	13 (5.0)	0.623^(^2^)^
Previous stroke		152 (5.5)	16 (6.1)	0.689^(^2^)^
COPD		195 (7.1)	14 (5.4)	0.292^(^2^)^
Peripheral artery insufficiency		121 (4.4)	25 (9.6)	<0.001^(^2^)^
Cerebrovascular disease		46 (1.7)	9 (3.1)	0.136^(^3^)^
Last creatinine level		1.3±0.7	1.3±0.8	0.498^(^1^)^
Previous MI		1267 (46.1)	143 (54.8)	0.008^(^2^)^
CHF		74 (2.7)	10 (3.8)	0.287^(^2^)^
Angina		2036 (74.1)	200 (76.6)	0.380^(^2^)^
Unstable angina		496 (18.1)	45 (17.2)	0.741^(^2^)^
EuroSCORE		2.7±3.1 (1.8)	2.7±3.2 (1.6)	0.031^(^4^)^
Nonelective surgery		21 (0.8)	7 (2.7)	0.008^(^3^)^
IABP		20 (0.7)	0 (0.0)	0.410^(^3^)^
CAD (>70%)	0*	534 (23.6)	45 (20.6)	
	1	355 (15.7)	44 (20.2)	0.322^(^2^)^
	2	731 (32.3)	71 (32.6)	
	3	644 (28.5)	58 (26.6)	
LMCAD (>70%)		182 (8.0)	21 (9.6)	0.412^(^2^)^
Severe proximal LMCAD		711 (31.4)	75 (34.4)	0.363^(^2^)^
Ejection fraction		63.7±12.8	63.3±12.71	0.718^(^1^)^

(1) Descriptive probability level of Student’s t-test.

(2) Descriptive probability level of chi-square test.

(3) Descriptive probability level of Fisher's exact test.

(4) Descriptive probability level of Mann-Whitney nonparametric test.

BMI=body mass index; CHF=chronic heart failure; COPD=chronic obstructive pulmonary disease; CVD=cardiovascular disease; EuroSCORE=European System for Cardiac Operative Risk Evaluation; IABP=intra-aortic balloon pump; LMCAD=left main coronary artery disease; MI=myocardial infarction; PCI=percutaneous coronary intervention

* In this case, the surgical team did not provide the catheterization film report for inclusion in the database.

**Table 4:** Preoperative clinical characteristics. Matched cohort.

Variable		(n=522)PCI		*P*-value
		**No.** **(n=261)**	**Yes** **(n=261)**	
Median age (years)		61.3±9.9	61.0±10	0.712^(^1^)^
Male sex		187 (71.7)	192 (73.6)	0.624^(^2^)^
Race	White	210 (80.5)	228 (87.4)	
	Black	14 (5.4)	7 (2.7)	0.156^(^3^)^
	Asians	33 (12.6)	24 (9.2)	
	Others	4 (1.5)	2 (0.8)	
BMI	<25	97 (37.9)	87 (34.8)	
	25-30	115 (44.9)	106 (42.4)	0.285^(^2^)^
	≥30	44 (17.2)	57 (22.8)	
Smoking	Previous	107 (41.0)	107 (41.0)	
	Never	107 (41.0)	123 (47.1)	0.111^(^2^)^
	Current	47 (18.0)	31 (11.9)	
Family history of CVD		76 (29.1)	80 (30.7)	0.702^(^2^)^
Diabetes mellitus		90 (34.5)	106 (40.6)	0.148^(^2^)^
Dyslipidemia		104 (39.9)	142 (54.4)	<0.001^(^2^)^
Chronic kidney failure		12 (4.6)	13 (5.0)	0.838^(^3^)^
Previous stroke		12 (4.6)	16 (6.1)	0.437^(^3^)^
COPD		15 (5.8)	14 (5.4)	0.849^(^2^)^
Peripheral artery insufficiency		10 (3.8)	25 (9.6)	0.009^(^2^)^
Cerebrovascular disease		6 (2.3)	8 (3.1)	0.588^(^3^)^
Last creatinine level		1.3±0.6	1.3±0.8	0.860^(^1^)^
Previous MI		143 (54.8)	143 (54.8)	1.000^(^2^)^
CHF		5 (1.9)	10 (3.8)	0.190^(^2^)^
Angina		203 (77.8)	200 (76.6)	0.754^(^2^)^
Unstable angina		51 (19.5)	45 (17.2)	0.498^(^2^)^
EuroSCORE		2.5±2.1 (1.7)	2.7±3.2(1.6)	0.108^(^4^)^
Nonelective surgery		1 (0.4)	7 (2.7)	0.068^(^3^)^
IABP		-	-	-
CAD (>70%)	0	55 (25.4)	45 (20.6)	
	1	43 (19.8)	44 (20.2)	0.702^(^2^)^
	2	65 (30.0)	71 (32.6)	
	3	54 (24.9)	58 (26.6)	
LMCAD (>70%)		16 (7.4)	21 (9.6)	0.398^(^2^)^
Severe proximal LMCAD		62 (28.6)	75 (34.4)	0.190^(^2^)^
Ejection fraction		65.2±13	63.3±12.7	0.252^(^1^)^

(1) Descriptive probability level of Student’s t-test.

(2) Descriptive probability level of chi-square test.

(3) Descriptive probability level of Fisher's exact test.

(4) Descriptive probability level of nonparametric Mann-Whitney test.

BMI=body mass index; CHF=chronic heart failure; COPD=chronic obstructive pulmonary disease; CVD=cardiovascular disease; EuroSCORE=European System for Cardiac Operative Risk Evaluation; IABP=intra-aortic balloon pump; LMCAD=left main coronary artery disease; MI=myocardial infarction; PCI=percutaneous coronary intervention

### Clinical Outcomes in 30 Days, 1 Year and 5 Years

The 30-day, 1-year and 5-year endpoints in the original and matched cohorts are shown in Tables 1 and 2. There is no difference in PCI and non-PCI groups regarding mortality of any cause and cardiovascular mortality at 30 days, 1 year and 5 years either in original and matched cohort. In the original cohort, there is a higher and statistically significant difference in readmission of any cause, cardiovascular readmission and composite endpoints of cardiovascular death/readmission in the 1-year follow-up. There was also a higher, statistically significant difference between readmission of any cause and cardiovascular readmission in the 5-year follow-up. In the paired cohort, there is no statistically significant difference in any of these outcomes.

**Fig. 1:**
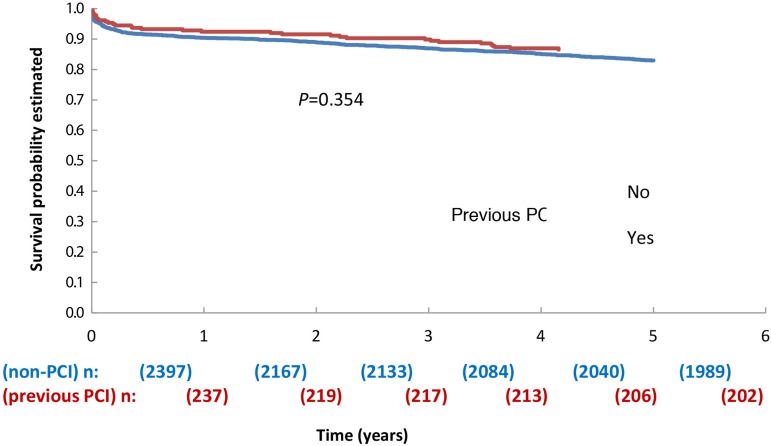
Kaplan-Meier actuarial survival curve.

The Kaplan-Meier actuarial survival curve (Figure 1) shows that there is no difference between "previous PCI" and "no previous PCI" groups along the 5-year follow-up.

## DISCUSSION

The profile of patients presenting for CABG has changed along the decades. Patients are older, with comorbidities and submitted to previous interventions, especially PCI. The indication of PCI to treat coronary artery disease has progressively increased in patients with multivessel disease, left main lesions including cases with complex coronary anatomy that are candidates for surgery according to US and European guidelines. The number of multiples PCI procedures has also increased. Consequently, the number of patients presenting for CABG with previous PCI increased significantly. The percentage of patients with previous PCI has been reported in the literature to vary from 13 to 40%. In this particular study, it is 8.7%.

Since 2005, single center or multicenter, regional or registry studies have investigated the influence of previous PCI on CABG regarding early, medium term or long term mortality and some of them have also studied influence on morbidity^[1-29]^.

In 2005, Hassan et al.^[4]^, in the analysis of 632 patients from 2 centers in Canada, showed a strong correlation of previous PCI and in-hospital mortality with an odds ratio (OR) of 1.93; *P*=0.003. In the following year, Thielmann et al.^[5]^ studied 2626 patients at Essen University in Germany and found that PCI increases mortality and adverse MACCE after CABG. The authors also showed that this influence was stronger after multiple PCIs. On the other hand, in 8 centers and 37140 patients in northwest Germany, Massoudy et al.^[7]^ have shown that 2 or more PCIs are strongly associated to mortality and MACCE either in a multivariate logistic regression as well as in a propensity score matching comparison. In a previous study by our group at the Heart Institute of the University of São Paulo with 1099 patients, published in 2012, in the original cohort and in the propensity score matching cohort, PCI was correlated with in-hospital mortality^[1]^. Other reports have shown adverse effects of prior PCI on CABG. Pliam et al.^[8]^, in a single center study, observed increased in-hospital mortality and decreased 60-day survival in patients with more than 3 stents. In the same type of study, Tran et al.^[17]^ have shown increased immediate mortality and complications and decreased 2-year survival for previous PCI patients. Sakaguchi et al.^[28]^ have shown that multiple PCIs decrease survival and increase long-term cardiovascular events. Negargar et al.^[24]^, in another single center study, observed a negative influence of prior PCI on immediate post-CABG complications. Kinoshita et al.^[18]^, in a more especific series of diabetic patients who underwent off-pump CABG, found an increase in early mortality in the PCI group. In a single center off-pump CABG series, Carnero-Alcázar et al.^[27]^ demonstrated lower survival and MACCE-free survival at medium term follow-up for PCI patients.

Eifert et al.^[22]^, in a series of 200 patients, demonstrated increased early mortality and morbidity and lower 8-year survival. Manancio et al.^[30]^, in a series of 7855 patients of 4 Italian centers, demostrated higher early mortality and complications and lower 5-year survival. In a study by the Virginia State Registry (US), covering 99% of cardiovascular operations in this state, Mehta et al.^[23]^ analyzed 34316 patients and found no influence of PCI on early mortality; however, it was an independent predictor of major complications. Finally, Ueki et al.^[20]^ conducted the first meta-analysis colecting data of comparative studies of previous PCI and no previous PCI until April 2014. In 23 studies and 174,777 patients, the authors showed that PCI increases the OR of hospital mortality (1.187). Futhermore, a subgroup analysis by proportion of multiple PCI suggests that multiple PCI further increases mortality.

On the other hand, several authors in diferent types of studies have found no negative influence of previous PCI on mortality and morbidity. Thus, van den Brule, Noyez and Verheugt^[9]^ did not observe influence in early mortality and several complications, as well as on 1-year follow-up. Gaszewska-żurek et al.^[16]^ reported that previous PCI did not affect CABG outcomes, but in this group freedom of angina is less likely. In the same way, Fukui et al.^[11]^ reported that previous PCI did not influence mortality and morbidity and in patients with postoperative angiography did not affect graft patency. Boening et al.^[13]^, in a single center study with diabetic patients, reported no influence on the risk of CABG. Bonaros et al.^[29]^, in a single center study, concluded that EuroSCORE and STS score are inacurate to predict early mortality in patients with previous PCI. A study of the Massachusetts State database and propensity score matching in previous PCI with primary CABG group found no negative influence of PCI on early and long-term mortality and adverse outcomes. In a study of the Spanish Ministry of Health database of 78,794 patients, 4.6% of them with previous PCI, Sanchez et al.^[19]^, in a univariate and multivariate analysis, as well as in a propensity score matching comparison, concluded that previous PCI was not an independent predictor of in-hospital mortality. Biancari et al.^[21]^, in a sistematic review and meta-analysis published in 2014, which included 9 studies and 68,645 patients, found that the PCI group has increased early mortality and morbidity. However, there was no influence in late mortality. Finally, Mariscalco et al.^[26]^ conducted the first prospective study of the European Multicenter Study on Coronary Artery Bypass Grafting (E-CABG) at 16 European centers, in a total of 3641 patients, of which 685 (19%) patients had a history of PCI. The study, published in 2018, showed that prior PCI was not associated with an increased risk of mortality or other adverse outcomes in patients undergoing CABG.

In the present study, we aimed to evaluate the influence of previous PCI on CABG in a single center in a series of 3007 consecutive patients followed for 30 days, 1 year and 5 years. In the preoperative clinical variables, in the original cohort, the comparison of 2746 patients without previous PCI and 261 with previous PCI, there were significant differences in age and frequency of dyslipidemia, peripheral artery disease, EuroSCORE and nonelective surgery. After propensity score matching of 261 patients, dyslipidemia and peripheral artery disease remained different. Regarding the outcomes of the present study, there was no significant influence of PCI on mortality at 30 days, 1 year and 5 years in either the original cohort or the matched cohort. In relation to other endpoints, there was a negative influence of PCI in readmission and composite endpoint death/cardiovascular readmission rate in the 1-year follow-up and readmission of any cause and cardiovascular readmission rate at 5-year follow-up. Neverthless, in the matched cohort, these differences were not confirmed. Therefore, in the present study, considering all comparisons, the impact of previous PCI on mortality and morbidity of CABG was not demostrated.

Possible factors affecting outcomes of CABG in previous PCI patients have been studied by some authors^[1-3,14,15]^. These include the occlusion of side branchs by the stent, causing microinfarction; an acute and chronic inflammatory process in the coronary artery wall caused by the stent itself and new-generation stent drugs; the presence of stent may obligate surgeons to perform anastomosis in a more distal and thinner position of coronary arteries. Consequently, the possibility of graft occlusion is greater. The stent can cause damage to the coronary artery wall and compromise the vasomotor response.

The present study has positive aspects and some limitations. The advantages are a good number of casuistry, single center, complete evaluation and follow-up of up to 5 years.

### Limitations

The retrospective nature of study; the lack of information on the precise interval between PCI and CABG; the lack of information about the number of PCI interventions and number of stents, and the difference between previous PCI and primary CABG groups even after propensity score matching.

## CONCLUSION

This particular analysis, there was no definitive negative influence of previous PCI on the mortality and morbidity of a future CABG surgery.

**Table:** 

Authors’ roles & responsibilities	
GSVM	Substantial contributions to the conception or design of the work; or the acquisition, analysis, or interpretation of data for the work; drafting the work or revising it critically for important intellectual content; final approval of the version to be published
AGS	Substantial contributions to the conception or design of the work; or the acquisition, analysis, or interpretation of data for the work; final approval of the version to be published
GSS	Substantial contributions to the conception or design of the work; or the acquisition, analysis, or interpretation of data for the work; final approval of the version to be published
FCC	Substantial contributions to the conception or design of the work; or the acquisition, analysis, or interpretation of data for the work; final approval of the version to be published
NAGS	Agreement to be accountable for all aspects of the work in ensuring that questions related to the accuracy or integrity of any part of the work are appropriately investigated and resolved; final approval of the version to be published
